# Corticosteroids Improve Renal Survival: A Retrospective Analysis From Chinese Patients With Early-Stage IgA Nephropathy

**DOI:** 10.3389/fmed.2020.585859

**Published:** 2020-10-22

**Authors:** Aiya Qin, Gaiqin Pei, Yi Tang, Li Tan, Xingfu Wei, Zhengxia Zhong, Ling Zhou, Changyun Chen, Wei Qin

**Affiliations:** ^1^Division of Nephrology, Department of Medicine, West China Hospital, Sichuan University, Chengdu, China; ^2^West China School of Medicine, Sichuan University, Chengdu, China; ^3^Institute of Epidemiology and Health Statistics, School of Public Health, Lanzhou University, Lanzhou, China; ^4^Affiliated Hospital of Zunyi Medical College, Zunyi, China; ^5^The Third Hospital of Zigong City, Zigong, China; ^6^People's Hospital of Mianzu, Mianzu, China

**Keywords:** immunoglobulin A nephropathy (IgAN), immunosuppressive therapy, corticosteroids, supportive care, renal survival

## Abstract

**Background:** The efficacy and safety of corticosteroids and immunosuppressive therapy remain controversial for the treatment of immunoglobulin A nephropathy (IgAN). This study aimed to evaluate the effects of corticosteroid and immunosuppressant therapy in Chinese patients with early-stage IgAN whose estimated glomerular filtration rate (eGFR) was ≥45 ml/min/1.73 m^2^ and proteinuria was ≥1 g/24 h at biopsy.

**Methods:** Patients with biopsy-proven IgAN were retrospectively enrolled from four study centers between 2007 and 2016. Patients were regularly followed up for at least 1 year or until the study end point. Patients were categorized into three treatment groups: supportive care (SC), steroids alone (CS), and steroids plus immunosuppressants (IT). The observed responses to therapy included complete remission (CR), partial remission (PR), no response (NR), and end-stage renal disease (ESRD). The primary end point of the current study was defined as a 50% decline in eGFR and/or ESRD.

**Results:** A total of 715 patients (male 47% and female 53%) were recruited and followed up for 44.69 ± 24.13 months. The observed CR rate was 81.8% with corticosteroids alone (CS), 62.7% with corticosteroids + immunosuppresants (IT), and 37% with supportive care alone (SC). Renal outcomes were remarkably better in the CS group compared with the SC and IT groups (the percentage of patients reaching the end point in each group was 4.6 vs. 14.4 vs. 11.5%, respectively; *p* = 0.001). Moreover, 36 and 80-month renal survival were significantly better for the CS group (98.3 and 86.4%) than for the IT (94.2 and 82.4%) and SC (94.0 and 51.6%) groups. Early CKD stage also presented with better kidney survival (*p* < 0.001). Renal survival of CKD stage 1 patients was relatively good regardless of the specific treatment regimen. CS and IT treatment significantly improved renal survival for CKD stage 2 patients when compared with the SC group (*p* < 0.001 and 0.007, respectively). However, renal survival of CKD stage 3a patients was not impacted by any of the three treatment regimens. Subgroup analysis also showed that renal survival of patients with proteinuria >3.5 g, M1, E0, S1, T0, and C0 was significantly better in the CS group than in the SC and IT groups. A multivariate model showed that hypertension, serum creatinine, E1 lesion, and T1/T2 lesion remained independent predictors of poor renal survival.

**Conclusions:** Immunosuppressive therapy does not have further benefit beyond that provided by steroids. Corticosteroids plus optimal supportive care may further be beneficial in treating early-stage IgAN patients in that it could significantly improve the short-term renal outcome.

## Introduction

Immunoglobulin A nephropathy (IgAN) is the most commonly observed type of glomerulonephritis (GN) worldwide. It is characterized by the presence of IgA immune complex deposits in the mesangium of kidney (1). As many as 20–40% of IgAN patients will progress to end-stage renal disease (ESRD) within 20 years from the time of diagnosis (2). The best treatment plan for IgAN patients is still controversial. The 2012 Kidney Disease Improving Global Outcome (KDIGO) guidelines suggested the use of corticosteroids in patients with estimated glomerular filtration rate (eGFR) >50 ml/min per 1.73 m^2^ and persistent proteinuria >1 g/day that has not responded to 3–6 months of optimized supportive care with renin–angiotensin system blockers (3). However, several trials have shown discordant results for this treatment plan. The STOP-IgAN trial found that corticosteroids alone or corticosteroids plus cyclophosphamide did not reduce the decrease rate of eGFR compared to the control group (4). Others have reported that corticosteroids and immunosuppressive treatment can improve short-term renal outcome in advanced-stage IgAN patients, but not their long-term prognosis (5). In order to shed additional light on this controversial issue, we compared the effect of supportive care, corticosteroids alone, and corticosteroids + immunosuppressants in a cohort of Chinese patients with early-stage IgAN (eGFR ≥ 45 ml/min/1.73 m^2^ and mean proteinuria ≥ 1 g/24 h) in the current study.

## Materials and Methods

### Patients

Patients with IgAN confirmed by renal biopsy with any additional systemic diseases (systemic lupus erythematosus, diabetes mellitus, Henoch–Schönlein purpura, liver cirrhosis, etc.) were retrospectively enrolled from across four study centers (West China Hospital of Sichuan University, Zunyi Medical College Affiliated Hospital, The Third People's Hospital of Zigong City, and People's Hospital of Mianzhu City) between December 2007 and February 2016. Patients with eGFR ≥ 45 ml/min/1.73 m^2^ and proteinuria ≥ 1 g/24 h at biopsy were included. All patients were followed for at least 12 months or until the study reached its pre-defined end point. Treatment was chosen according to patients' willingness and doctors' experience. Written informed consent was obtained from all patients. The study adhered to the Helsinki Declaration and was approved by the Ethics Committee of West China Hospital of Sichuan University (2019-33).

The 2012 KDIGO guideline suggested to use corticosteroids in patients with eGFR >50 ml/min per 1.73 m^2^ and persistent proteinuria >1 g/day despite 3–6 months of optimized supportive care. However, the role for corticosteroids is limited to cases with minimal change on light microscopy in IgAN with nephrotic syndrome, or glomerular filtration rate is impaired severely or there are pathologic features of chronic injury in crescentic IgAN. Since there were no standard treatment guidelines for patients presented with significantly heterogeneous clinical and pathological manifestation, therapeutic regimens were determined by both the doctors' experience and the patients' willing based on current consensus. Adverse drug events were carefully described to each patient. Patients were given optimal supportive therapy if they refused to take steroids or immunosuppressants for steroid-related adverse effects. Patients were categorized into three groups according to the chosen therapy strategies. Patients in the supportive care group (SC) only received an optimal dose of an angiotensin-converting enzyme inhibitor (ACEI) or an angiotensin receptor blocker (ARB). Patients in the corticosteroids group (SC) received optimal ACEI/ARB plus corticosteroids (0.8–1 mg/kg/day prednisone or an equivalent dose of methylprednisolone for 2–3 months, then tapered by 20% every 2 weeks for the next 6–8 months). Patients in the immunosuppressant group (IT) received the same dose of corticosteroids plus immunosuppressant therapy (2 mg/kg/day cyclophosphamide for 3 months, or 1–2 g/day mycophenolate mofetil for 6–8 months). Because calcineurin inhibitors (CNI) are not yet recommended in IgAN patients, we excluded patients treated with cyclosporin A (CsA) or tacrolimus (FK). In addition, patients with tonsillectomy or those using fish oil were excluded from this study.

### Data Collection

We recorded sex, age, systolic blood pressure (SBP), diastolic blood pressure (DBP), serum creatinine (Scr), eGFR, serum albumin (Alb), uric acid (UA), 24-h proteinuria level, and kidney pathology data for every patient. eGFR was calculated using the Chronic Kidney Disease Epidemiology Collaboration (CKD-EPI) equation. Follow-up duration is defined as the interval between renal biopsy and the last outpatient visit, death, or ESRD. Renal biopsies were evaluated by an experienced pathologist and a nephrologist. Lesion scores were classified according to the Oxford Classification of IgAN (6): mesangial score <0.5 or >0.5 (M0/M1); segmental glomerulosclerosis absent or present (S0/S1); endocapillary hypercellularity absent or present (E0/E1); tubular atrophy/interstitial fibrosis <25%, 26–50%, or >50% (T0/T1/T2); and cellular or fibrocellular crescents absent or in at least 1 glomerulus or in at least 25% of glomeruli (C0/C1/C2).

### Definitions of Clinical Responses and Outcome

Responses to therapy included complete remission (CR), partial remission (PR), no response (NR) or ESRD. CR is defined as a reduction of proteinuria to <0.5 g/24 h and a decline in eGFR by at least 10% from the baseline. PR is defined as a reduction in proteinuria ≥50% and in eGFR by at least 10% from the baseline. NR is defined as a reduction in proteinuria by at least 50%, or a decline in eGFR by at least 10% from the baseline. ESRD is defined as eGFR <15 ml/min/1.73 m^2^, chronic dialysis, or renal transplantation. The combined end point is defined by a renal function decline >50% in eGFR and/or ESRD.

### Statistical Analyses

Categorical variables are expressed as frequencies and percentages and then were compared using Fisher and chi-squared tests. Continuous variables are presented as mean ± SD and were analyzed by *t*-test, one-way ANOVA, or Kruskal–Wallis *H*-test, as appropriate. Kidney survival in each group was estimated by the Kaplan–Meier method, and survival curves were compared by the Log-rank test. Univariate and multivariate Cox proportional hazard models were used to evaluate the influence of clinical and pathological variables on renal outcomes. IBM SPSS Statistics 22.0 was used to perform all statistical analyses and *p* < 0.05 was considered significant.

## Results

### Baseline Characteristics

**Figure 1:**
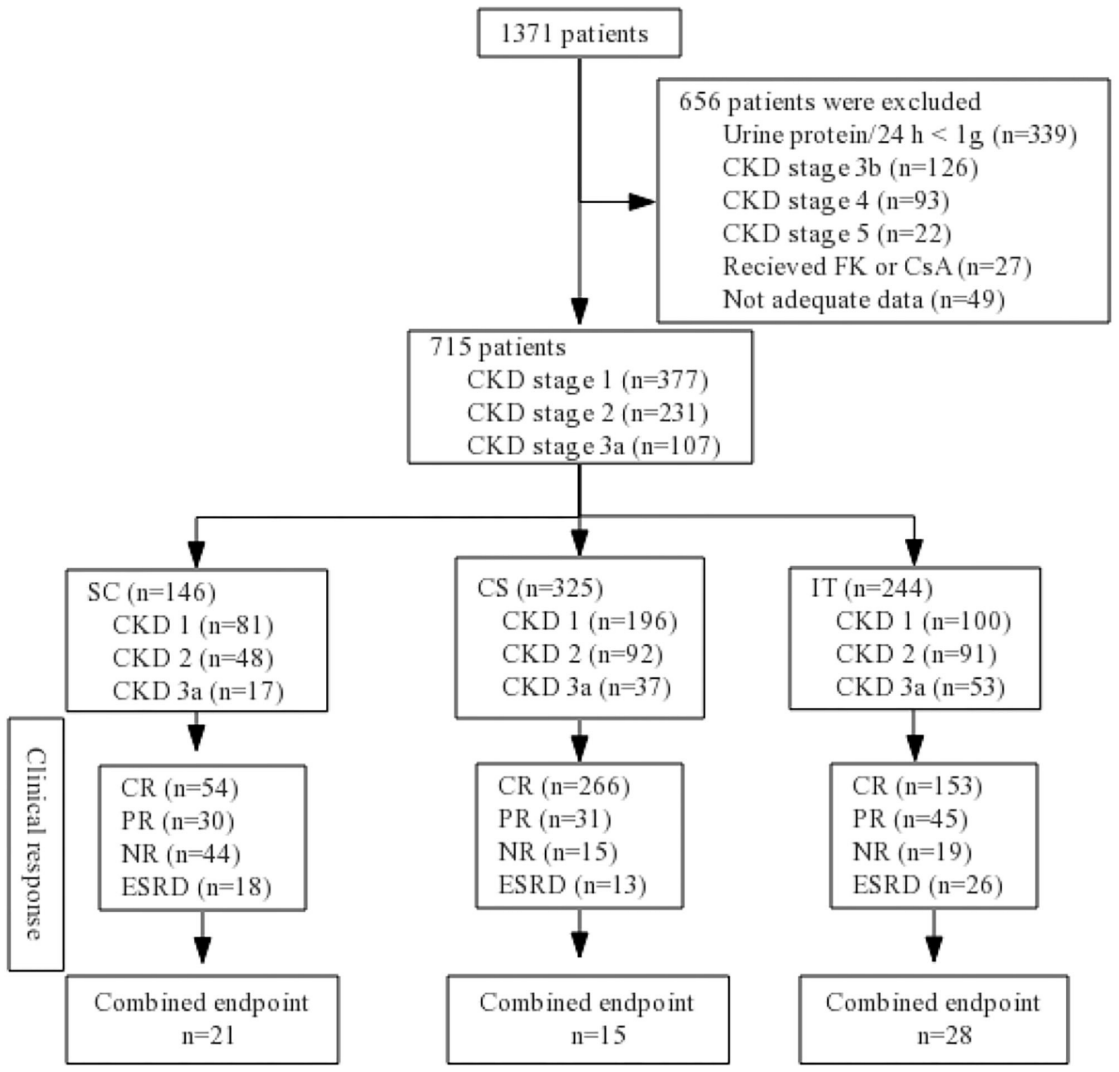
Flow diagram. CKD, chronic kidney disease; SC, supportive care group; CS, corticosteroids; IT, immunosuppressive therapy; CR, complete remission; PR, partial remission; NR, no response; ESRD, end-stage renal disease; FK, tacrolimus; CsA, cyclosporin A.

A total of 1,371 patients were screened, and from them, 656 were excluded according to the exclusion criteria: urine protein/24 h <1 g (*n* = 339); CKD stage 3b (*n* = 126); CKD stage 4 (*n* = 93); CKD stage 5 (*n* = 22); receiving treatment with CsA or FK (*n* = 27); inadequate data available (*n* = 49). In all, 715 biopsy-confirmed IgAN patients (male 47% and female 53%) were recruited into this study (Figure 1). The basic characteristics of the 715 enrolled patients at baseline are shown in Table 1. Patients were followed for 44.69 ± 24.13 months on average. Of the patients, 146 (20.42%) received supportive care (SC), 325 (45.45%) received corticosteroids (CS), and 244 (34.13%) were treated with both immunosuppressant and corticosteroids (IT). The baseline analyses indicated that patients in the IT group presented with significantly more severe clinical and pathological manifestations, such as higher Scr, lower eGFR, greater 24-h proteinuria, higher SBP, and severe renal pathologic changes compared to patients in the SC and CS groups (Table 1). There were no baseline differences among three groups in age, sex, hypertension, DBP, or UA. Characteristics according to different CKD stages are presented in Supplementary Table 1. In CKD 1 patients, hypertension, urine protein, and pathologic lesions (M, E, S, T, and C) were significantly higher in IT group than those in SC and CS groups. Less proteinuria, higher Alb, lower rate of nephrotic syndrome, and M1/C1/C2 were observed in the SC group in CKD 2 patients. There were no significant differences among three groups in the CKD 3 stage at baseline analysis.

**Table 1:** Baseline clinicopathological characteristics of IgAN patients in different therapies.

**Characteristics**	**Groups**			***p-*value**
	**SC (*n* = 146)**	**CS (*n* = 325)**	**IT (*n* = 244)**	
Follow-up (months)	44.69 ± 24.13			
**Clinical**				
Male gender (%)	69 (47.3)	151 (46.5)	116 (47.5)	0.996
Age (years)	34.29 ± 10.24	32.15 ± 11.36	33.52 ± 11.33	0.113
Hypertension (%)^a^^b^	41 (28.1)	95 (29.2)	95 (38.9)	0.023
Nephrotic syndrome (%)^a^^c^	2 (1.4)	90 (27.7)	53 (21.7)	<0.001
SBP (mmHg)^b^	129.51 ± 18.56	126.37 ± 18.56	133 ± 20.18	<0.001
DBP (mmHg)	83.19 ± 14.06	81.86 ± 13.10	84.68 ± 14.83	0.058
Serum creatinine (μmol/L)^a^^b^	86.37 ± 27.55	83.28 ± 26.64	95.23 ± 28.82	<0.001
eGFR (ml/min per 1.73 m^2^)^b^	93.86 ± 26.99	100.95 ± 71.77	85.04 ± 28.44	0.002
Urine protein (g/24 h)^a^^c^	1.74 ± 0.876	3.61 ± 3.15	3.71 ± 2.62	<0.001
Serum albumin (g/L)^a^^c^	40.49 ± 4.08	35.06 ± 8.79	36.42 ± 7.00	<0.001
Uric acid (μmol/L)	368.42 ± 96.91	359.21 ± 101.64	374.95 ± 94.85	0.163
CKD stage			<0.001	
Stage 1 (%)	81 (55.5)	196 (60.3)	100 (41.0)	
Stage 2 (%)	48 (32.9)	92 (28.3)	91 (37.3)	
Stage 3a (%)	17 (11.6)	37 (11.4)	53 (21.7)	
**Pathologic (Oxford classification)**				
M1 (%)^a^^b^	108 (74.0)	244 (75.1)	211 (86.5)	0.001
E1 (%)^a^^c^	1 (0.7)	17 (5.2)	23 (9.4)	0.001
S1 (%)^a^^b^	74 (50.7)	154 (47.4)	156 (63.9)	<0.001
T1/T2 (%)^a^^b^	23 (15.8)	45 (13.8)	63 (25.8)	0.001
C1/C2 (%)^a^^b^^c^	17 (11.6)	77 (23.7)	86 (35.2)	<0.001

*Data presented as number (percentage) or mean ± SD. eGFR, estimated glomerular filtration rate; CKD, chronic kidney disease; SBP, systolic blood pressure; DBP, diastolic blood pressure; M, mesangial proliferation; E, endocapillary proliferation; S, segmental sclerosis; T, tubular atrophy/interstitial fibrosis; C, crescents; SC, supportive care group; CS, corticosteroids; IT, immunosuppressive therapy*.

a *Stands for p < 0.05 between SC and IT*.

b *Stands for p < 0.05 between CS and IT*.

c *Stands for p < 0.05 between SC and CS*.

### Treatment Response and Outcome

Following treatment, 473 patients (66.2%) achieved CR, 106 patients (14.8%) achieved PR, 78 patients (10.9%) showed NR, and 57 patients (8.0%) ended in ESRD during the treatment follow-up period. One patient (0.1%) in the IT group died of severe pulmonary infection (Table 2). We observed that patients in the CS group (266 patients, 81.8%) had a significantly higher CR rate than patients in either the IT group (153 patients, 62.7%) or the SC group (54 patients, 37%; *p* < 0.001). Conversely, the NR and ESRD rates were remarkably higher in the SC group (*p* < 0.001 and *p* = 0.001, respectively). At the end of follow-up, 64 patients (9.0%) reached the combined end point. Most of these patients were in the SC group (21, 14.4%) or IT group (28, 11.5%; *p* = 0.01). As shown in Table 2, patients in the CS group had an apparently higher total remission rate (CR + PR; 91.3%) and a lower poor renal outcome rate (4.6%) compared with those in the SC (57.5 and 14.4%) and IT (81.1 and 11.5%) groups.

**Table 2:** Response and outcome.

**Parameter**	**All patients** ***n* = 715**	**Therapy groups**			***p*-value**
		**SC** ***n* = 146**	**CS** ***n* = 325**	**IT** ***n* = 244**	
**Response**					
CR	473 (66.2)	54 (37.0)	266 (81.8)	153 (62.7)	<0.001
PR	106 (14.8)	30 (20.5)	31 (9.5)	45 (18.4)	0.001
NR	78 (10.9)	44 (30.1)	15 (4.6)	19 (7.8)	<0.001
ESRD	57 (8.0)	18 (12.3)	13 (4.0)	26 (10.7)	0.001
Death	1 (0.1)	0 (0.0)	0 (0.0)	1 (0.4)	1.000
**CKD 1**					
CR	287 (76.1)	37 (45.7)	178 (90.8)	72 (72.0)	<0.001
PR	51 (13.5)	23 (28.4)	12 (6.1)	16 (16.0)	<0.001
NR	30 (8.0)	19 (23.5)	4 (2.0)	7 (7.0)	<0.001
ESRD	9 (2.4)	2 (2.5)	2 (1.0)	5 (5.0)	0.090
**CKD 2**					
CR	142 (61.5)	16 (33.3)	71 (77.2)	55 (60.4)	<0.001
PR	37 (16.0)	5 (10.4)	12 (13.0)	20 (22.0)	0.129
NR	28 (12.1)	17 (35.4)	6 (6.5)	5 (5.5)	<0.001
ESRD	24 (10.4)	10 (20.8)	3 (3.3)	11 (12.1)	0.003
**CKD 3a**					
CR	44 (41.1)	1 (5.9)	17 (45.9)	26 (49.1)	0.005
PR	18 (16.8)	2 (11.8)	7 (18.9)	9 (17.0)	0.940
NR	20 (18.7)	8 (47.1)	5 (13.5)	7 (13.2)	0.010
ESRD	24 (22.4)	6 (35.3)	8 (21.6)	10 (18.9)	0.363
Death	1 (0.9)	0 (0.0)	0 (0.0)	1 (1.9)	1.000
**Outcome**					
Combined end point	64 (9.0)	21 (14.4)	15 (4.6)	28 (11.5)	0.001
50% decline in eGFR	56 (7.8)	18 (12.3)	14 (4.3)	24 (9.8)	0.004
ESRD	57 (8.0)	18 (12.3)	13 (4.0)	26 (10.7)	0.001
Death	1 (0.1)	0 (0)	0 (0)	1 (0.4)	1.000

*Data presented as number (percentage). CKD, chronic kidney disease; CR, complete remission; eGFR, estimated glomerular filtration rate; PR, partial remission; NR, no response; ESRD, end-stage renal disease; SC, supportive care group; CS, corticosteroids; IT, immunosuppressive therapy*.

For stage 1 CKD patients, the CR rate was significantly higher for patients in the CS group (CS 90.8 vs. IT 72.0 vs. SC 45.7%; *p* < 0.001). The incidence of ESRD was similar between the CS, IT, and SC treatment groups (1.0, 5.0, and 2.5%, respectively; *p* = 0.090). In patients with stage 2 CKD, there was an apparent difference in the CR rate (CS 77.2 vs. IT 60.4 vs. SC 33.3%; *p* < 0.001) and ESRD rate (CS 3.3 vs. IT 12.1 vs. SC 20.8%; *p* = 0.003). However, in patients with stage 3a CKD, the ESRD rate was similar across groups (IT 18.9 vs. CS 21.6 vs. SC 35.3%; *p* = 0.363).

### Renal Survival

**Figure 2:**
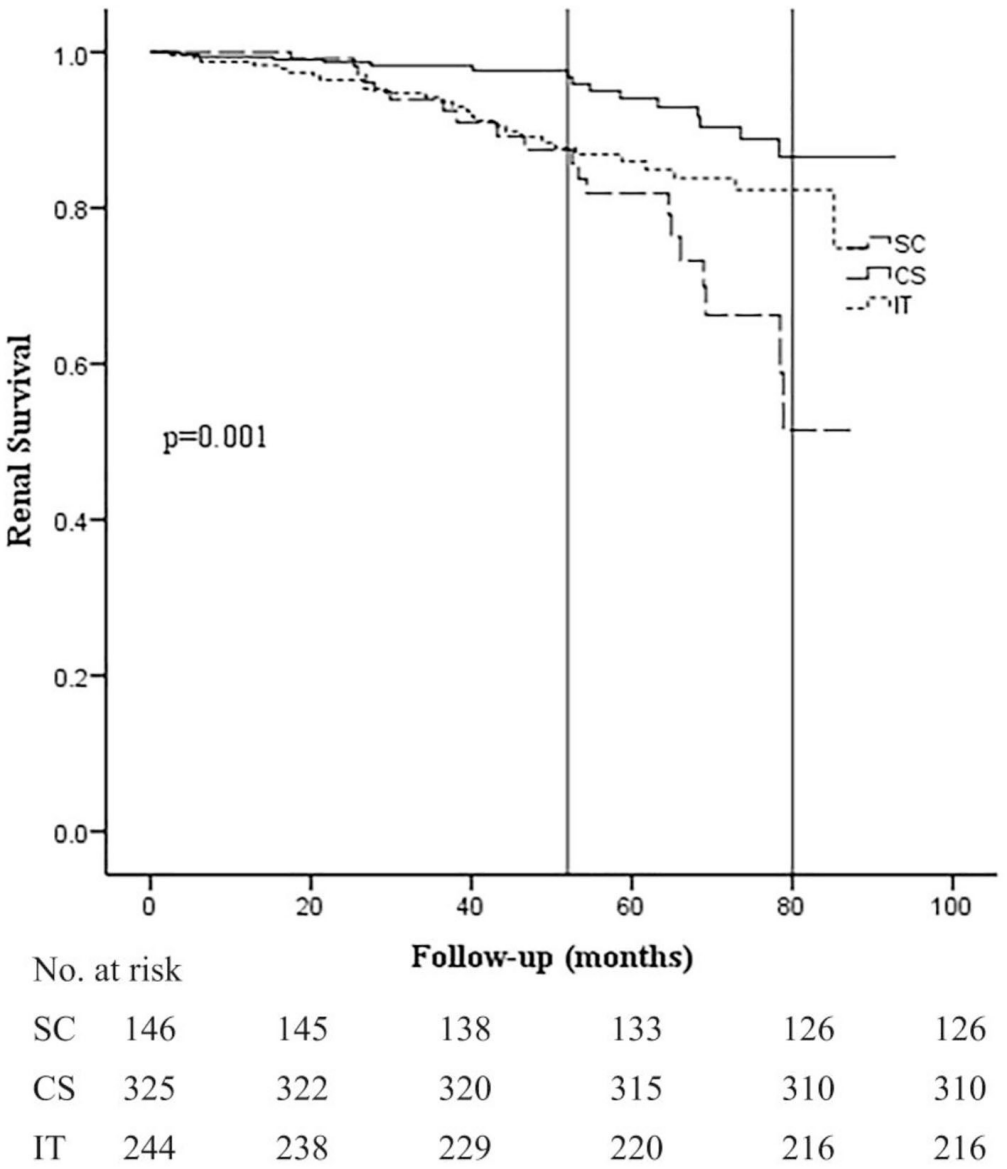
Kaplan–Meier analysis for the probability of composite end point in the SC, CS, and IT group. The composite end point was 50% decline in eGFR and/or ESRD. SC, supportive care group; CS, corticosteroids; IT, immunosuppressive therapy.

Kaplan–Meier analysis revealed that renal survival during the follow-up period was significantly better in the CS group at 36, 60, and 80 months (98.3, 94.0, and 86.4%) compared to the SC (94.0, 82.1, and 51.6%) and IT groups (94.2, 85.8, and 82.4%; *p* = 0.001; Figure 2). Differences in renal survival between the SC and IT groups were observed in short-term follow-up (36 months, *p* = 0.048) but not in long-term follow-up (60 and 80 months, *p* = 0.926 and 0.127). It is worth noting that the survival curve of the CS group remained stable for about 52 months and then decreased rapidly thereafter, whereas survival curve of the IT group decreased rapidly in the beginning and became relatively stable after 52 months. At the end of the follow-up period, the survival curve in the CS and IT groups almost overlapped.

**Figure 3:**
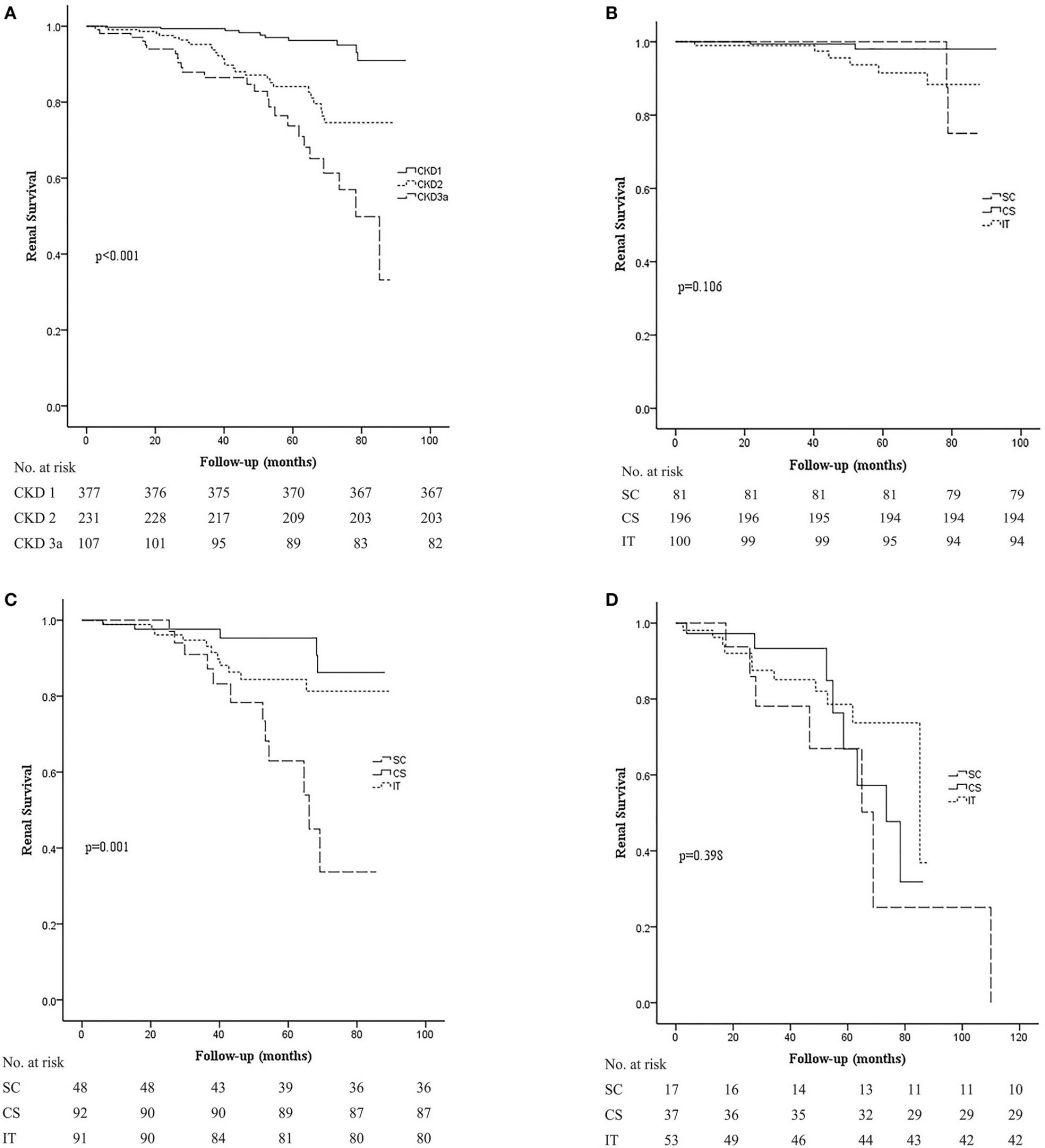
Kaplan–Meier analysis for the probability of composite end point in different CKD stages. The composite end point was 50% decline in eGFR and/or ESRD. **(A)** Kidney survival rates in CKD stage 1, 2, and 3a groups. **(B)** Kidney survival rates in the SC, CS, and IT group in patients in the CKD stage 1 group. **(C)** Kidney survival rates in the SC, CS, and IT group in patients in the CKD stage 2 group. **(D)** Kidney survival rates in the SC, CS, and IT group in patients in the CKD stage 3a group. SC, supportive care group; CS, corticosteroids; IT, immunosuppressive therapy.

When comparing the kidney survival rates, we noticed that early-stage CKD disease presented with better kidney survival (*p* < 0.001; Figure 3A). Renal survival of stage 1 CKD patients was relatively good regardless of the different treatment regimens (Figure 3B). In stage 2 CKD patients, treatment with corticosteroids alone (CS) or along with immunosuppressants (IT) improved renal survival rate compared to supportive care alone (SC; *p* < 0.001 and 0.007). Nevertheless, we found no statistically significant difference between the CS and IT groups (*p* = 0.219; Figure 3C), which indicated that the addition of an immunosuppressant may not be necessary for these patients. For stage 3a CKD patients, renal survival rate was relatively poor overall, and unfortunately even aggressive treatment with both corticosteroids and immunosuppressants did not improve renal outcome in these patients (*p* = 0.398; Figure 3D). These results suggest that optimal supportive care may be the best choice for patients with moderate stage IgAN.

Subgroup analysis of proteinuria, M, E, S, T, and C for the probability of composite end point by Kaplan–Meier analysis is shown in Table 3. In patients with proteinuria ≤3.5 g, the rate of renal survival declined from the CS group, the IT group, to the SC group (94.1 vs. 90.9 vs. 88.4%), but only statistically significant difference could be found between CS and SC groups (*p* = 0.035). Renal survival of patients with proteinuria >3.5 g, M1, E0, S1, T0, and C0 was significantly better in the CS group than in the SC and IT groups. These clinical and morphology data determined that these patients should be treated with steroids.

**Table 3:** Subgroup analysis of clinical and morphology data for the probability of composite end point by Kaplan-Meier analysis.

**Parameter**	***N*** **of events/total (% of renal survival)**			***p*****-value**		
	**SC**	**CS**	**IT**	**SC vs. CS**	**SC vs. IT**	**CS vs. IT**
Proteinuria≤3.5 g	16/138 (88.4)	12/205 (94.1)	13/143 (90.9)	0.035	0.126	0.663
Proteinuria >3.5 g	5/8 (37.5)	3/120 (97.5)	15/101 (85.1)	<0.001	0.001	0.002
M0	2/38 (94.7)	0/81 (100.0)	0/33 (100.0)	0.032	0.148	/
M1	19/108 (82.4)	15/244 (93.9)	28/211 (86.7)	<0.001	0.075	0.047
E0	21/145 (85.5)	13/308 (95.8)	24/221 (89.1)	<0.001	0.052	0.024
E1	0/1 (100.0)	2/17 (88.2)	4/23 (82.6)	0.808	0.835	0.692
S0	8/72 (88.9)	7/171 (95.9)	6/88 (93.2)	0.073	0.205	0.676
S1	13/74 (82.4)	8/154 (94.8)	22/156 (85.9)	<0.001	0.094	0.022
T0	11/123 (91.1)	3/280 (98.9)	10/181 (94.5)	<0.001	0.040	0.017
T1/T2	10/23 (56.5)	12/45 (73.3)	18/63 (71.4)	0.140	0.147	0.806
C0	17/129 (86.8)	11/248 (95.6)	17/158 (89.2)	0.001	0.164	0.066
C1/C2	4/17 (76.5)	4/77 (94.8)	11/86 (87.2)	0.004	0.110	0.177

*M, mesangial proliferation; E, endocapillary proliferation; S, segmental sclerosis; T, tubular atrophy/interstitial fibrosis; C, crescents; SC, supportive care group; CS, corticosteroids; IT, immunosuppressive therapy*.

### Risk Factor of Renal Survival Predictors

The correlations between clinicopathological parameters and renal end point were analyzed using a Cox regression model (Table 4). In a univariate analysis, gender, eGFR, Scr, hypertension, M1, E1, S1, T1/T2, and CKD stage at the time of biopsy were all factors that were significantly associated with renal survival. A multivariate Cox regression analysis further showed that hypertension (HR 1.99, 95% CI 1.16–3.42; *p* = 0.012), Scr (HR 1.02, 95% CI 1.00–1.05; *p* = 0.024), E1 (HR 3.10, 95% CI 1.14–8.42; *p* = 0.027), and T1/T2 (HR 3.34, 95% CI 1.98–6.33; *p* < 0.001) remained as independent predictors of renal survival.

**Table 4:** Cox proportional hazard model for the primary end point in IgA nephropathy patients.

**Risk factor**	**Univariable**		**Multivariable**	
	**HR (95% CI)**	***p*-value**	**HR (95% CI)**	***p*-value**
**Gender**				
Male	0.56 (0.34–0.93)	0.025	1.36 (0.62–2.98)	0.445
Female	1 (Referent)			
Hypertension	3.96 (2.39–6.56)	<0.001	1.99 (1.16–3.42)	0.012
Urinary protein	1.05 (0.98–1.13)	0.192	1.08 (0.98–1.18)	0.109
Serum creatinine	1.03 (1.02–1.04)	<0.001	1.02 (1.00–1.05)	0.024
eGFR	0.96 (0.95–0.97)	<0.001	1.00 (0.99–1.01)	0.943
CKD 1	1 (Referent)			
CKD 2	4.96 (2.41–10.21)	<0.001	1.62 (0.64–4.11)	0.309
CKD3a	9.71 (4.66–20.24)	<0.001	0.80 (0.20–3.26)	0.759
M1	6.14 (1.50–25.16)	0.012	3.19 (0.75–13.52)	0.116
E1	2.33 (1.00–5.41)	0.051	3.10 (1.14–8.42)	0.027
S1	2.32 (1.36–3.94)	0.002	1.51 (0.86–2.65)	0.155
T1/T2	8.87 (5.30–14.83)	<0.001	3.34 (1.98–6.33)	<0.001
C1/C2	1.41 (0.82–2.41)	0.213	0.81 (0.43–1.53)	0.521
Age	0.996 (0.97–1.02)	0.781		
Serum albumin	0.99 (0.96–1.02)	0.507		
NS	1.00 (0.55–1.85)	0.991		

*HR, hazard ratio; 95% CI, 95% confidence interval; eGFR, estimated glomerular filtration rate; CKD, chronic kidney disease; M, mesangial proliferation; E, endocapillary proliferation; S, segmental sclerosis; T, tubular atrophy/interstitial fibrosis; C, crescents*.

## Discussion

IgAN is the most commonly diagnosed primary GN, especially in Asia. Previous studies of treatments for IgAN patients have yielded discordant results. Currently, RASI, corticosteroids, and immunosuppressive therapy are commonly used in clinical practice. Although the KDIGO guidelines provide general recommendations for the treatment of IgAN, the guidelines do not take pathological changes and ethnic differences into account (3, 7). Indeed, it is difficult to remain completely consistent with the guidelines in clinical practice. Due to the various individual clinicopathological characteristics of patients and the possibility of severe adverse events, the treatment plans chosen by doctors and patients are often different. There are currently no large clinical trials underway to evaluate the efficacy of different treatments for early-stage IgAN patients. Some studies have found that the addition of immunosuppressive therapy in treatment plans for IgAN patients did not significantly improve renal outcome, while several other studies have suggested the opposite (4, 5, 8, 9). Thus, further studies with large sample sizes and long-term follow-up are critically needed to estimate the effects of different treatment regimens and predict the best therapeutic regimens.

In this study, we enrolled 715 patients from four study centers and followed up for 44.69 ± 24.13 months. Our results showed that corticosteroids alone had a significant effect on slowing progression to ESRD for early-stage IgAN patients (especially in stage 1 CKD). Steroid treatment was better than added immunosuppressant therapy or supportive care along in achieving CR (81.8 vs. 62.7 vs. 37.0%) and a kidney survival (CS 98.3 and 86.4% vs. IT 94.2 and 82.4% vs. SC 94.0 and 51.6% at 36 and 80 months, respectively; *p* < 0.001). These results provide new evidence for the use of corticosteroids alone in patients with early-stage IgAN. Currently, corticosteroid use in patients with IgAN is inconsistent, and it is difficult to summarize a personalized treatment (7). The KDIGO guidelines based on RCTs recommend the use of CS in high-risk individuals with IgAN with eGFR >50 ml/min/1.73 m^2^ and proteinuria >1 g/day despite 6 months of optimized supportive therapy. However, the evidence supporting this guideline is low level (2C) (3). Recently, the STOP-IGAN trial and VALIGA cohort study both indicated that there is no significant difference in renal survival between corticosteroids plus renin–angiotensin system blockades (RASBs) vs. RASBs alone (4, 8). However, several other studies found that the use of corticosteroids improved outcomes compared with control groups (5, 9), especially in Asian patients. It is worth noting that results from the TESTING trial showed that oral methylprednisolone might improve renal outcome and decrease eGFR decline in Chinese patients with IgAN. Results from the current study demonstrated that corticosteroids can significantly increase clinical remission rates and improve renal survival in Chinese IgAN patients with eGFR ≥45 ml/min/1.73 m^2^ and proteinuria ≥1 g/24 h during a mean follow-up period of 44.69 months. Subgroup analysis also showed that renal survival of patients with CKD 2 stage, proteinuria >3.5 g, M1, E0, S1, T0, and C0 was significantly better in the CS group than in the SC and IT groups. These clinical and morphology data support the idea that these patients should be treated with steroids. It was important to note that the survival curve of the CS group remained almost stable for about 52 months before decreasing rapidly thereafter. These results indicate that the short-term effects of corticosteroids are better than the long-term effects. One possible mechanism for this might be that the early application of steroids can inhibit inflammation, immune responses, and fibrosis of the kidney, resulting in improved renal prognosis. Nevertheless, the reactivation of inflammation and immune responding after cessation of the steroid treatment could subsequently lead to poorer long-term prognosis. Further research is needed to confirm this hypothesis.

We also found that patients in the IT group had significantly higher Scr, lower eGFR, higher 24-h proteinuria, higher SBP, and more severe renal pathologic changes than the SC and CS groups in the current study. Though the KDIGO guidelines does not currently recommend immunosuppressive therapies for IgAN patients, they are sometimes used in clinical practice for patients with high-risk and active pathological changes. Immunosuppressive drugs are used to modulate the immune response, inhibit inflammation, relieve fibrosis and mesangial proliferation, and reduce levels of galactose-deficient IgA1 (10). However, the use of immunosuppressant drugs for IgAN is in dispute due to the difficulty in balancing between toxicity and long-term renal survival (11). Some reports found that immunosuppressants might lower proteinuria and improve renal outcome in patients with IgAN (12, 13), while several studies did not find prominent benefits from an immunosuppressive combination protocol (14, 15). Our study indicated that renal survival was significantly better in the CS group than in the IT group during the follow-up period. More severe clinical manifestations (lower eGFR and higher proteinuria) and pathological changes (more M, S, T, and C lesions) in patients in the IT group may explain why poorer renal outcome was observed more in patients in the IT group than in the CS group. This outcome indicated that immunosuppressive therapy did not result in further benefit beyond steroids. In addition, the renal survival curve for the IT group decreased rapidly at the beginning while becoming relatively stable after 52 months, which suggests that immunosuppressants may be beneficial for long-term renal survival in IgAN patients. However, these findings must be validated by further study.

Results from the current study showed that the effect of treatment was largely dependent on patients' baseline eGFR levels. In stage 1 CKD patients, renal survival was considerable despite the different treatment regimens. In stage 2 CKD patients, patients treated with either CS or IT had better renal survival than patients treated with SC alone. In contrast, none of the treatment regimens provided good renal survival in stage 3a CKD IgAN patients. The results showed that corticosteroids could significantly increase the clinical remission and improve renal survival in Chinese IgAN patients with CKD2 and not CKD3a as shown in Figure 3D. This finding is in line with previous reports (12) that corticosteroids or immunosuppressants could only improve short-term renal outcome for advanced-stage IgAN patients. Based on the current findings, we recommend that treatment of IgAN should be initiated as early as possible.

In the Cox multivariate analysis, it was found that hypertension, Scr level, endocapillary hypercellularity, and tubular atrophy/interstitial fibrosis were significant independent predictors of poor renal outcome. These results are similar to previous reports (12, 16). Although several reports (17) have shown that patients with cellular or fibrocellular crescents have worse prognoses, crescents were not found to be associated with renal survival in the current study. This may be due to differences in the inclusion criteria between the studies. It is clear that large-sample clinical trials are needed before conclusions can be drawn. Previous studies have also shown that the amount of proteinuria is an established risk factor in IgAN (18), but proteinuria was not identified as a significant risk factor even in the univariate analysis in this study. This may be explained by the fact that we only included patients with proteinuria >1 g/day.

Although we reported several interesting and novel results in this study, there are study limitations that should be noted. First, this is a retrospective, single ethnicity (Chinese Han) study. We have to admit that the baseline characters of patients in different groups were not matched in this study. The underlying reason of this difference may be the treatment choice of doctor. Doctors tend to choose more aggressive treatment (corticosteroids or immunosuppressants) in patients with severer clinical and pathological features. Therefore, this observational study could only reflect the exact effect of different treatment in real clinical practice environment, so results from this study may not be generalizable to patients from other regions of the world. A well-designed controlled study may provide more information. Second, the follow-up period was relatively short. Considering that IgAN is a slow progressive disease, a much longer follow-up period (more than 10 years) may be needed to reach more credible conclusions. Thirdly, one patient in the IT group (CKD 3a stage) died of severe pulmonary infection in this study. No other severe adverse effects were recorded. It seems that most IgAN patients were tolerable to corticosteroids and immunosuppressive therapy. No more severe adverse event was reported by patients. Most of the patients could not remember minor or moderate adverse events when we collect information from them. Therefore, further large-scale, multicenter studies with long-term follow-up and more detailed clinical and pathological data should be undertaken to provide more scientific justification for the best treatment plans for patients with IgAN.

## Conclusion

Immunosuppressive therapy does not have further benefit beyond that provided by steroids. Corticosteroids plus optimal supportive care may further be beneficial in treating early-stage IgAN patients in that there is significant improvement of the short-term renal outcome. Treatment of IgAN should also be initiated as early as possible, especially at CKD stage 1.

## Data Availability Statement

The raw data supporting the conclusions of this article will be made available by the authors, without undue reservation.

## Ethics Statement

The studies involving human participants were reviewed and approved by The Ethics Committee of West China Hospital of Sichuan University. The patients/participants provided their written informed consent to participate in this study.

## Author Contributions

AQ, GP, YT, and WQ: conception and design. AQ, GP, LT, and ZZ: administrative support. AQ, GP, LT, ZZ, LZ, CC, and WQ: collection and assembly of data. AQ, GP, YT, LT, XW, ZZ, LZ, CC, and WQ: data analysis and interpretation. GP, AQ, YT, LT, XW, ZZ, LZ, CC, and WQ: manuscript writing. AQ, GP, YT, LT, XW, ZZ, LZ, CC, and WQ: final approval of manuscript. All authors contributed to the article and approved the submitted version.

## Conflict of Interest

The authors declare that the research was conducted in the absence of any commercial or financial relationships that could be construed as a potential conflict of interest. The reviewer FL has declared a shared affiliation with several of the authors AQ, GP, YT, LT, and WQ, to the handling editor at the time of review.
