# Supplementary material for: Corticosteroids Improve Renal Survival: A Retrospective Analysis From Chinese Patients With Early-Stage IgA Nephropathy
Source: Front Med (Lausanne). 2020 Oct 22;7:585859. doi: 10.3389/fmed.2020.585859 (PMC7643022; doi:10.3389/fmed.2020.585859)
Supplement: Supplementary file 1 [file Table_1.DOC]

**Sup 1.** **Baseline clinicopathological characteristics of IgAN patients in different CKD stages.**

Data presented as number (percentage) or mean ± SD. eGFR, estimated glomerular filtration rate; CKD, chronic kidney disease; M, mesangial proliferation; E, endocapillary proliferation; S, segmental sclerosis; T, tubular atrophy/interstitial fibrosis; C, crescents; SC, supportive care group; CS, corticosteroids; IT, immunosuppressive therapy; a stands for p < 0.05 between SC and CS; b stands for p < 0.05 between SC and IT; c stands for p < 0.05 between CS and IT;

| Characteristics | Groups | | | P value |
| --- | --- | --- | --- | --- |
|  | SC | CS | IT |  |
| **CKD 1** | | | |  |
| Male | 36(44.4%) | 91(46.4%) | 47(47.0%) | 0.943 |
| Age (year) | 31.19±9.60 | 29.03±9.73 | 29.99±11.59 | 0.271 |
| Hypertension ^bc^ | 13(16.0%) | 42(21.4%) | 39(39.0%) | <0.001 |
| Nephrotic syndrome ^ab^ | 0(0.0%) | 64(32.7%) | 26(26.0%) | <0.001 |
| Serum creatinine (μmol/L) | 69.38±13.09 | 68.39±15.62 | 71.91±16.49 | 0.177 |
| Urine protein (g/24h) ^ab^ | 1.54±0.55 | 3.57±2.52 | 4.06±3.10 | <0.001 |
| Serum albumin (g/L) ^ab^ | 40.84±3.91 | 33.92±9.29 | 35.49±8.36 | <0.001 |
| M1 ^c^ | 60(74.1%) | 139(70.9%) | 82(82.0%) | 0.117 |
| E1 ^bc^ | 0(0.0%) | 9(4.6%) | 13(13.0%) | <0.001 |
| S1 ^c^ | 40(49.4%) | 72(36.7%) | 59(59.0%) | 0.001 |
| T1/T2 ^c^ | 6(7.4%) | 11(5.6%) | 13(13.0%) | 0.070 |
| C1/C2 ^abc^ | 4(4.9%) | 44(22.4%) | 34(34.0%) | 0.117 |
| **CKD 2** |  |  |  |  |
| Male | 22(45.8%) | 40(43.5%) | 41(45.1%) | 0.955 |
| Age (year) ^b^ | 38.15±9.37 | 36.63±12.15 | 34.02±10.15 | 0.076 |
| Hypertension | 20(41.7%) | 33(35.9%) | 35(38.5%) | 0.786 |
| Nephrotic syndrome ^ab^ | 1(2.1%) | 18(19.6%) | 18(19.8%) | 0.012 |
| Serum creatinine (μmol/L) | 96.22±16.91 | 95.68±17.27 | 99.80±17.21 | 0.233 |
| Urine protein (g/24h) ^ab^ | 1.85±1.09 | 3.73±4.30 | 3.61±2.36 | 0.002 |
| Serum albumin (g/L) ^ab^ | 40.51±4.29 | 37.19±8.11 | 37.05±6.06 | 0.008 |
| M1 ^b^ | 34(70.8%) | 78(84.8%) | 81(89.0%) | 0.020 |
| E1 | 1(2.1%) | 6(6.5%) | 6(6.6%) | 0.536 |
| S1 | 24(50.0%) | 57(62.0%) | 58(63.7%) | 0.256 |
| T1/T2 | 9(18.8%) | 20(21.7%) | 25(27.5%) | 0.468 |
| C1/C2 ^b^ | 8(16.7%) | 24(26.1%) | 33(36.3%) | 0.043 |
| **CKD 3a** |  |  |  |  |
| Male | 11(64.7%) | 20(54.1%) | 28(52.8%) | 0.677 |
| Age (year) | 38.24±10.96 | 37.54±11.91 | 39.32±10.37 | 0.747 |
| Hypertension | 8(47.1%) | 20(54.1%) | 21(39.6%) | 0.409 |
| Nephrotic syndrome | 1(5.9%) | 8(21.6%) | 9(17.0%) | 0.390 |
| Serum creatinine (μmol/L) | 51.16±4.22 | 52.45±3.86 | 51.96±4.91 | 0.316 |
| Urine protein (g/24h) | 2.44±1.096 | 3.50±2.90 | 3.274±1.99 | 0.272 |
| Serum albumin (g/L) | 38.81±4.12 | 35.81±6.55 | 37.10±5.43 | 0.192 |
| M1 | 14(82.4%) | 27(73.0%) | 48(90.6%) | 0.081 |
| E1 | 0(0.0%) | 2(5.4%) | 4(7.5%) | 0.853 |
| S1 | 10 (58.8%) | 25(67.6%) | 39(73.6%) | 0.497 |
| T1/T2 | 8(47.1%) | 14(37.8%) | 25(47.2%) | 0.652 |
| C1/C2 | 5(29.4%) | 9(24.3%) | 19(35.8%) | 0.518 |
